# A Comparison between Mucoderm® and Connective Tissue Graft for Root Coverage

**DOI:** 10.30476/DENTJODS.2021.90830.1535

**Published:** 2022-09

**Authors:** Alireza Fathiazar, Roya Shariatmadar Ahmadi, Ferena Sayar

**Affiliations:** 1 Dept. of Periodontics, Faculty of Dentistry, Ardabil University of Medical Sciences, Ardabil, Iran; 2 Dept. of Periodontics, Faculty of Dentistry, Tehran Medical Sciences, Islamic Azad University, Tehran, Iran

**Keywords:** Gingival Recession, Connective tissue, Collagen matrix

## Abstract

**Statement of the Problem:**

Subepithelial connective tissue graft (SCTG) is the gold stand-ard treatment for root coverage procedure; however, this technique has limitations such as the need for a donor site and the difficulty of the harvesting procedure. The potential bene-fits of Mucoderm®, a collagen matrix derived from porcine dermis, as an alternative treat-ment for root coverage can be investigated.

**Purpose:**

This study aimed to evaluate the efficacy of Mucoderm® for root coverage and compare its results with SCTG.

**Materials and Method:**

This double-blind split-mouth randomized clinical trial was con-ducted on seven patients with 12 bilateral gingival recessions (24 recession sites). Coronally advanced flap + Mucoderm® was applied on one side and coronally advanced flap + con-nective tissue graft (CTG) was applied on the contralateral side. We measured the periodon-tal pocket depth (PPD), clinical attachment level (CAL), recession depth (RD), keratinized tissue width (KTW) and gingival thickness (GT) with a surgical stent at baseline (preopera-tively) and at 1, 3 and 6 months postoperatively. The Wilcoxon and Friedman tests were used to analyse the data.

**Results:**

The mean percentage of root coverage was 26% in the Mucoderm® group and 60% in the SCTG group at 6 months, compared with baseline. The mean percentage of root coverage was significantly different between the two groups (*p* Value< 0.05). The results indicated that Mucoderm® did not increase the KTW, while CTG significantly increased the KTW (*p* Value< 0.05 at 1, 3 and 6 months).

**Conclusion:**

The results of this study showed that Mucoderm® might not be an appropriate alternative for the CTG in root coverage procedures.

## Introduction

Gingival recession refers to apical migration of the gingival margin relative to the cementoenamel junction (CEJ) [ 1 ]. Exposure of the root surface may lead to an unaesthetic appearance, root hypersensitivity, and difficult oral hygiene maintenance. No direct relationship has been reported between gingival recession and tooth loss; however, the progression of buccal gingival recession may compromise the longevity of the tooth [ 2 ].

Several techniques have been recommended for root coverage such as the pedicle and free autogenous grafts [ 3 ]. At present, subepithelial connective tissue graft (SCTG) is considered as the gold standard for the root coverage procedure [ 4 ]. Successful results have been reported using SCTG especially for Miller’s class I and II recessions [ 5 ]. However, it has some restrictions such as the need for a secondary surgical procedure in the palate to harvest the graft and limited amount of tissue to harvest [ 6 ]. To overcome these limitations, allografts and xenografts were introduced to the market [ 7 ].

Collagen matrix as an alternative for connective tissue graft (CTG) has been proposed for root coverage procedure in different studies. It has been mentioned that the use of a collagen matrix instead of the CTG could reduce the time of surgery and the pain that the patients suffer during and after surgery [ 1 , 3 - 4 ]. However, there are contradicting results regarding the root coverage percentage by using collagen matrices in comparison with the CTG. According to similar studies, the root coverage percentage using collagen matrices have been reported to be in the range of 64% to 96% [ 3 - 4 ].

Mucoderm® (Botiss, Germany) is a xenogeneic collagen matrix derived from porcine. Following the purification procedures, a 3D matrix comprising of types I and type III collagen is produced with a structure similar to that of connective tissue [ 8 ]. Only a few studies have evaluated the efficiency of Mucoderm® as an alternative to CTG in periodontal plastic surgery [ 7 , 9 ] Therefore, this study aimed to evaluate the efficacy of Mucoderm® for root coverage compared with the CTG.

## Materials and Method

### Patient selection and preparation

This study was a double blind, split-mouth randomized clinical trial, which was conducted in Department of Periodontics, Faculty of Dentistry, Islamic Azad University, Tehran, Iran. Seven patients with 12 bilateral Mille-r’s class I and II gingival recessions (24 recession sites) participated in this study. Five patients had multiple recessions and two had bilateral single recession sites.

The patients willingly signed informed consent forms prior to their participation in the study. This study was approved by the institute review committee for human subjects with code number (IR.IAU.DENTAL. REC.1397.023) and the human subjects ethics board of the Iranian registry of clinical trials (IRCT code: IRCT-20140318017053N10) and was conducted in accordance with the Helsinki Declaration of 1975, as revised in 2013.

The patients were non-smokers, did not have systemic or periodontal diseases, were not pregnant or lactating, and did not use medications with adverse effects on the gingiva. The recession sites around decayed teeth, crowns, and orthodontic wires were also excluded from the study. Oral hygiene instructions were provided and non-surgical periodontal treatments were performed to decrease the O'Leary’s plaque index of patients below 20%. Patients were also instructed to avoid traumatic tooth brushing.

The study parameters including periodontal pocket depth (PPD), clinical attachment level (CAL), keratinized tissue width (KTW), gingival thickness (GT) and recession depth (RD) were measured at baseline (preoperatively) and at 1,3and6 months postoperatively.

PPD was measured from the gingival margin to the bottom of the sulcus, and CAL was measured from the CEJ to the bottom of the sulcus at the midbuccal aspect of the tooth [ 10 ]. RD was measured from the CEJ to the gingival margin, and KTW was measured from the gingival margin to the mucogingival junction in the midbuccal aspect of the teeth by using a Williams probe [ 11 ]. GT was determined as thin or thick biotype using the transgingival probing technique [ 12 ].

In order to avoid possible errors in consecutive measurements, one stent was fabricated for each patient, so the angulation and placement of periodontal probe would be more accurate. All parameters were measured by a periodontist (F.S) who was not aware of the test or control sides of the patients.

### Surgical techniques

**Figure 1:**
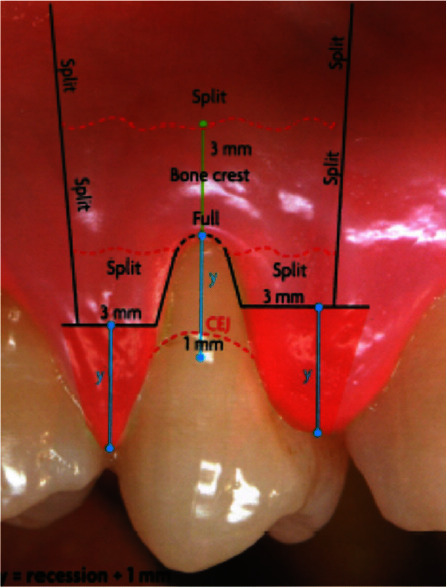
A schematic illustration of incisions

According to a computer-generated randomization list (Microsoft Excel 2010), one side was treated by Mucoderm® and the contralateral side was treated with CTG. Local anesthesia was administered using lidocaine plus 1:100,000 epinephrine (Persocaine, Darupakhsh, Iran) [ 13 ]. The incision was made according to the Zucchelli’s bilaminar coronally advanced flap technique [ 14 ]. The incision was made using a #15 carbon steel scalpel (Moris, Germany). One sulcular incision and then two horizontal incisions (Figure 1) were made from the base of the mesial papilla to the base of the distal papilla. The horizontal incisions did not reach the adjacent tooth (the vertical distance from the tip of the papilla to the horizontal incision was equal to gingival recession + 1mm). Two divergent vertical incisions were made corono-apically to 3-4mm beyond the mucogingival junction. A full-thickness flap was elevated apical to the recession site, and split-thickness flaps were elevated at the mesial and distal parts of the recession site. Thus, a trapezoidal flap design was prepared. The exposed root surface was debrided and root-planed, and the adjacent papillae were deepithelialized. The muscle tensions were released and an appropriate bed was prepared to serve as the recipient site [ 14 ] (Figure 1).

**Figure 2:**
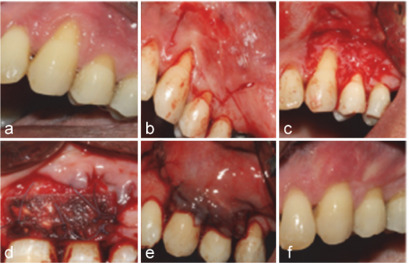
A multiple recession site treated with coronally advanced flap and the Mucoderm®; **a:** Two teeth with gingival recessions before surgical procedure, **b:** Vertical and horizontal incisions are made, **c:** The partial thickness flap is elevated, **d:** Mucoderm is placed and sutured, **e:** The partial thickness flap is sutured, **f:** The result is shown after 6 months

In the test group, Mucoderm® (Botiss Dental Company, Germany) was applied as a graft for root coverage (the Mucoderm® thickness was about 1.2 to 1.7mm). In the control group, the connective tissue harvested from the palate by the envelope technique [ 15 ] was used as the root coverage graft. The length of the connective tissue graft depended on the recipient site, and the thickness of the graft was about 1mm [ 14 ]. The grafts were fixed to the adjacent tissues with interrupted sutures at the level of the CEJ (Vicryl, Supa, Iran). The flaps were coronally advanced and fixed by interrupted sutures (Vicryl, Supa, Iran) [ 11 ] (Figures 2 [Fig JDS-23-402-g003.tif] [Fig JDS-23-402-g004.tif] to  5).

**Figure 3 JDS-23-402-g003.tif:**
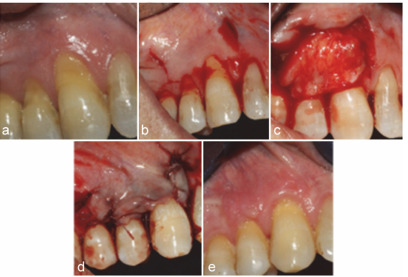
A multiple recession site in the same patient as in figure1, which is treated with coronally advanced flap and the connective tissue graft; **a:** Two teeth with gingival recessions before surgical procedure, **b:** Vertical and horizontal incisions are made, **c:** The Connective Tissue Graft is placed, **d:** The partial thickness flap is sutured, **e:** The result is demonestrated after 6 months

**Figure 4 JDS-23-402-g004.tif:**
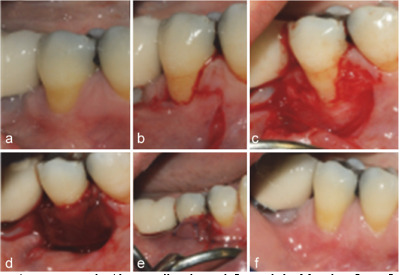
A single recession area treated with coronally advanced flap and the Mucoderm®; **a:** One tooth with gingival recession before surgical procedure, **b:** Vertical and horizontal incisions are made, **c:** The partial thickness flap is elevated, **d:** Mucoderm is placed and sutured, **e:** The partial thickness flap is sutured, **f:** The result is shown after 6 months

**Figure 5:**
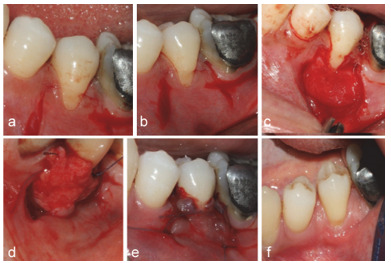
A single recession area in the same patient as in figure 3, which is treated with coronally advanced flap with the connective tissue graft; **a:** One tooth with gingival recession before surgical procedure, **b:** Vertical and horizontal incisions are made, **c:** The partial thickness flap is elevated, **d:** Connective Tissue is placed, **e:** The partial thickness flap is sutured, **f:** The result is shown after 6 months

### Post-surgical considerations

Amoxicillin (500mg; Tehranshimi, Iran) was prescribed 3 times a day for 6 days, and ibuprofen (600 mg; Aria, Iran) was prescribed twice a day for 1 week [ 1 ]. The sutures were removed after 2 weeks. The patients were instructed to avoid tooth brushing for 4 weeks and 0.2% chlorhexidine mouth rinse (Shahrdaru, Tehran) was prescribed twice daily [ 16 ].

The patients’ plaque index, PPD, CAL, KTW, GT and RD were measured at 1, 3 and 6 months, postoperatively. The mean percentage of root coverage after 6 months was calculated using the following formula: Mean root coverage= (RD (baseline)-RD(T6)/ RD baseline) ×100[ 11 ].

### Statistical analysis

To calculate the minimum sample size, we conducted a pilot study on four patients, and t-test on the mean root coverage values after 1 month was performed. The results revealed that a minimum of nine samples were required considering α=0.05, β=0.2 and standard deviation of 34%. 

The data were analyzed by Freedman (within-group) and Wilcoxon (between-group) tests. SPSS version 25 (SPSS Inc., IL, USA) was used for statistical analysis, and *p* Value<0.05 was considered statistically significant.

## Results

Seven patients participated in this clinical trial. One patient had bilateral multiple recessions in mandibular canines and lateral incisors. Two patients had bilateral single recessions in mandibular premolars. Two patients had bilateral multiple recessions at the site of maxillary canines and premolars. One patient had multiple recessions at the site of mandibular canine and premolars, and one patient had bilateral recessions at the site of mandibular canines.

The mean and standard deviation of PPD, CAL, RD, KTW, and root coverage are reported in Table 1. These values were calculated at baseline (T0, preoperatively), and at 1 (T1), 3 (T3) and 6 (T6) months, postoperatively.

**Table 1:** Mean (± standard deviation) PPD, CAL, RD, KTW, and RC in the test and control groups

Parameters	Mucoderm				SCTG			
	T0	T1	T3	T6	T0	T1	T3	T6
Pocket Probing Depth	1.17±0.38	1.75±0.62	1.17±0.38	1.17±0.38	1.25±0.45	2.17±0.93	1.25±0.45	1.33±0.49
Clinical Attachment Level	4.92±1.37	4.42±1.67	4±1.2	3.75±1.05	5.25±1.05	3.5±1	3.08±1	2.92±1.24
Recession Depth	3.83±1.11	2.75±1.65	2.83±1.11	2.75±1.13	3.92±1.08	1.25±0.96	1.67±1.07	1.58±0.99
Keratinized Tissue Width	1.58±1.83	2.42±2.23	1.58±1.44	1.17±1.11	1.33±1.43	4.25±2.73	3.75±2.22	3.33±1.87
Root Coverage Percentage		31±26%	24±21%	26±23%		64±26%	57±24%	60±22%

Table 2 shows the differences between the Mucoderm® and SCTG for PPD, CAL, RD, KTW, and root coverage using the Wilcoxon Signed Rank test. As shown in Table 2, there was no significant difference in any parameter between the Mucoderm® and SCTG groups at baseline (T0; *p*> 0.05).

**Table 2:** Comparison of parameters between the Mucoderm and SCTG groups using the Wilcoxon Signed Rank test

Time		T0	T1	T3	T6
Parameters/groups					
PPDM PPDC	*p*= 0.31	*p*= 0.12	*p*=0.56	*p*= 0.31
CALM CALC	*p*= 0.27	*p*=0.020	*p*<0.05	*p*=0.032
RDM RDC	*p*=0.073	*p*< 0.05	*p*<0.05	*p*< 0.05
KTWM KTWC	*p*= 0.46	*p*< 0.05	*p*<0.05	*p*< 0.5

PPDM: Pocket Probing Depth in Mucoderm group

PPDC: Pocket Probing Depth in Connective tissue group

CALM:Clinical Attachment Level in Mucoderm group

CALC: Clinical Attachment Level in Connective tissue group

RDM: Recession Depth in Muccoderm group

RDC: Recession Depth in Connective tissue group

KTWM: Keratinized Tissue Width in Mucoderm group

KTWC: Keratinized Tissue Width in Connective tissue group

The mean RD in the Mucoderm®+coronally advanced flap group was 3.83±1.11mm at baseline, which changed to 2.75±1.13 mm after 6 months. The mean RD in the SCTG group was 3.92±1.08mm at baseline and 1.58±0.99mm after 6 months. The mean RC was 1.08 mm in the Mucoderm®+coronally advanced flap and 2.34mm in SCTG group. As shown in Table 1, the mean percentage of root coverage was 26% in the Mucoderm®+coronally advanced flap and 60% in the CTG+ coronally advanced flap group; this difference was significant (*p*< 0.05). The KTW was 1.58±1.83mm at baseline and 1.17±1.11mm after 6 months in the Mucoderm®+coronally advanced flap group. Thus, the KTW decreased by 0.41mm in the Mucoderm®+ coronally advanced flap group. The KTW in the SCTG group increased from 1.33±1.43mm to 3.33±1.87mm; thus, there was about 2mm increase in KTW after 6 months and this difference was significant (*p*<0.05). 

Three patients had thin and four had thick biotype. The biotype of all recession sites (whether treated by Mucoderm® or CTG) changed to thick biotype after the surgical procedure. In other words, the patients who had thin gingival biotype acquired thick gingival biotype and those with thick gingival biotype remained the same after surgery.

## Discussion

This split-mouth study was performed to compare Mucoderm® with CTG for root coverage procedure. According to the results of this study, Mucoderm® + coronally advanced flap had inferior results to CTG + coronally advanced flap in terms of root coverage percentage and KTW.

In a systematic review, Amine *et al.* [ 17 ] concluded that SCTG was still the gold standard for root coverage surgery, and xenogeneic collagen matrix had inferior results to the CTG. It should be noted that xenogeneic collagen matrices have variable structures and, in some studies, they showed comparable results to CTGs [ 1 , 11 ]. For instance, McGuire *et al.* [ 18 ] reported comparable results for Mucograft® and CTG for root coverage. Thus, xenogeneic collagen matrices may show variable results depending on their process of production and structure.

Cardopoli *et al.* [ 1 ] and Chevalier *et al.* [ 11 ] did not find significant differences between the CTG and xenogeneic collagen matrix (Mucograft®) for root coverage. Mucoderm®, similar to Mucograft®, is a collagen matrix derived from porcine dermis [ 19 ]. However, in order to find an explanation for the poor results of Mucoderm® in this study in comparison with Mucograft®, we need to consider their different structures. Mucograft® has a bilayer structure. The outer layer is condensed and occlusive but the inner layer has a porous structure, which allows the ingrowth of blood clot and the surrounding tissues [ 19 ]. Whilst, Mucoderm® has a uniform 3D structure composed of collagen and elastin [ 19 ]. Probably, the inner porous layer of Mucograft® improves tissue integration and better root coverage.

Taba *et al.* [ 20 ] conducted a study in which CTG and Mucoderm® were compared in patients with bilateral class I or II Miller gingival recessions. The results showed that root coverage percentage was 62% in Mucoderm® group and 75% in CTG group after 3 months. According to the results of their study, almost twice more root coverage percentage was achieved by Mucoderm® compared to the results of our study. However, it should be noted that 62% coverage was gained in 3 months but due to probable shrinkage of the collagen matrix (Mucoderm®), the coverage percentage might reduce in longer period. In another study, Vedyaeva *et al.* [ 21 ] reported 81% of root coverage in Mucoderm® group in patients with multiple recessions by using the tunnel technique. Perhaps, the thickness of Mucoderm® could be a positive aspect in tunneling technique but in coronally advanced flap due to the increase tension, the high thickness would reduce root coverage percentage after surgery.

In an animal study by Barbeck *et al.* [ 22 ], histological assessment of the tissues surrounding the Mucoderm® revealed polynuclear giant cells and inflammation-dependent angiogenesis, but such inflammatory signs were not found in Mucograft® group. Such differences may explain the poor results of Mucoderm® in comparison with Mucograft®. According to our results, Mucoderm® was not capable of increasing the KTW after 6 months but the CTG significantly increased the KTW after 6 months (*p*<0.5).

Two systematic reviews concluded that the xenogeneic collagen matrix is capable of increasing the KTW comparable to the CTG [ 16 , 23 ]. It should be mentioned that Mucograft® was used in most cases in the abovementioned systematic reviews. Papi *et al.* [ 24 ] evaluated the efficacy of Mucoderm® as a soft tissue augmentation graft around implants in a pilot study. They reported an increase in KTW after 12 months. However, they used Mucoderm® only to create keratinized tissue and did not consider implant thread coverage.

In our study, three patients had thin gingival biotypes at the recession sites, but after surgery, both the Mucoderm® and CTG groups showed a conversion from thin to thick biotype. Thus, both Mucoderm® and the CTG were capable of increasing the gingival thickness.

Mucoderm® is a new collagen matrix and only a few clinical studies have evaluated its efficacy [ 19 , 25 ]. Thus, more studies are needed to assess the capability of this collagen matrix in periodontal plastic surgeries.

## Conclusion

According to the results of the present study, Mucoderm® might not be a good alternative to CTG to increase the mean percentage of root coverage and KTW; but Mucoderm® can increase the gingival thickness comparable to the CTG.

## Acknowledgement

The authors wish to thank the participants for their contribution in this study.

## Conflict of Interest

The authors declare that they have no conflicts of interests.
